# Comparison of one-stage versus two-stage procedure for the management of patients with rotator cuff tear and concomitant shoulder stiffness

**DOI:** 10.1186/s13018-019-1075-3

**Published:** 2019-02-07

**Authors:** Hongwu Zhuo, Jian Li

**Affiliations:** Fuzhou Second Hospital Affiliated to Xiamen University, No.47, Shang Teng Street, Cang Shan District, Fuzhou, 350007 China

**Keywords:** Rotator cuff, Repair, Stiffness, Arthroscopic surgery, Capsular release, Conservative treatment

## Abstract

**Background:**

To compare the clinical outcomes of one-stage and two-stage procedures for the management of patients with rotator cuff tear and concomitant shoulder stiffness.

**Methods:**

From December 2013 to June 2016, we recruited 42 consecutive patients with rotator cuff tear and concomitant shoulder stiffness. Twenty-two patients underwent a one-stage procedure, including arthroscopic capsule release and concomitant rotator cuff repair, within 2 weeks of the diagnosis. For the remaining twenty patients, conservative treatment for the recovery of range of motion (ROM) was initially performed before arthroscopic rotator cuff repair. The ROM, visual analogue scale (VAS), American Shoulder and Elbow Surgeons (ASES) score, Constant-Murley score, and satisfaction rate were assessed preoperatively; 3, 6, 12, and 24 months after surgery; and at final follow-up.

**Results:**

The mean follow-up period was 26.3 months (range, 24–33 months). No significant difference was noted in preoperative demographic data (age, sex, dominant/non-dominant, diabetes mellitus, thyroid disease, and duration of symptoms) between the two groups (*P* = 0.165, *P* = 0.580, *P* = 0.662, *P* = 0.716, *P* = 0.231, and *P* = 0.152, respectively). After treatment, all patients exhibited significant improvement in ROM and functional scores (*P* = 0.001 and *P* = 0.001, respectively). At 3 months postoperatively, the two-stage group exhibited significantly improved forward flexion and internal rotation compared with the one-stage group (*P* = 0.001 and *P* = 0.038, respectively). No significant difference in ROM was noted between the two groups at 6, 12, 24 months postoperatively and the final follow-up. In addition, no significant differences in VAS, ASES, Constant-Murley score and satisfaction rate were noted between the two groups at final follow-up (*P* = 0.319, *P* = 0.529, *P* = 0.711, and *P* = 0.085, respectively).

**Conclusion:**

In the treatment of rotator cuff tear with concomitant stiffness, although the recovery of ROM took longer in patients who underwent the one-stage procedure, satisfactory results at final follow-up can be achieved using either the one-stage procedure or two-stage procedure.

**Study design:**

Case-control study.

## Introduction

Rotator cuff tear is a common condition that causes shoulder pain and dysfunction in daily life [1, 2]. Many patients with rotator cuff tear might also exhibit concomitant shoulder stiffness with a reported incidence of up to greater than 40% [3, 4].

The optimal management of patients with rotator cuff tear and concomitant shoulder stiffness still remains controversial [5–14]. Traditionally, when a patient experiences a rotator cuff tear with concomitant shoulder stiffness, the stiffness should be addressed initially through conservative treatment prior to rotator cuff repair because the repair is a “shoulder-tightening” procedure and might increase the rate of postoperative shoulder stiffness [8–10]. However, this two-stage procedure would prolong patient’s suffering given the delay in surgery, and the rotator cuff tear might extend during the treatment for stiffness [5–7, 11–13]. A one-stage procedure was recently proposed for the management of patients with rotator cuff tears and concomitant stiffness [6, 11, 12]. However, to our knowledge, there are limited data comparing the clinical outcomes of one-stage and two-stage procedures for treatment of patients with rotator cuff tear and concomitant stiffness.

The purpose of the present study was to compare the clinical outcomes of one-stage and two-stage procedures for rotator cuff tear and concomitant stiffness. We hypothesized that no differences in range of motion (ROM), functional scores, and satisfaction rate at final follow-up would be noted between these two groups.

## Materials and methods

### Study population

This was a retrospective study. From December 2013 to June 2016, fifty-six consecutive patients with rotator cuff tear and concomitant shoulder stiffness underwent either one-stage or two-stage procedure for treatment at our institution. The inclusion criteria were as follows: (1) patients with a small-sized (tear size < 1 cm) or medium-sized (tear size 1~3 cm) full-thickness rotator cuff tear; (2) patients with a concomitant limited passive ROM: forward flexion was less than 100° passively, external rotation with the arm at the side was less than 30° passively, and internal rotation of a vertebral level where the thumb reached was lower than the first lumbar spine junction passively; (3) patients with a minimum follow-up period of 2 years. Patients who had previous shoulder fractures or previous surgical procedures on the ipsilateral joint were excluded. Patients with concomitant shoulder lesions, such as arthritis in the glenohumeral joint or labral lesions, were also excluded.

According to the criteria, forty-two patients were included in this study. There were 10 males and 32 females with a mean age of 54.1 years (range, 47–69 years). The mean follow-up period was 26.3 months (range, 24–33 months). The demographic data of the patients are summarized in Table 1. The one-stage group consisted of 22 patients, and the two-stage group consisted of 20 patients. Overall, no statistically significant differences in the demographic data (age, sex, dominant/non-dominant, diabetes mellitus, thyroid disease, and duration of symptoms) were noted between the groups (*P* = 0.165, *P* = 0.580, *P* = 0.662, *P* = 0.716, *P* = 0.231, and *P* = 0.152, respectively, Table 1). Approval of the study was obtained through the institutional review board at our institution (approval number, 10020151028). All patients provided signed informed consent to allow their clinical and radiologic data to be used for research programs.

**Table 1:** Demographic data^a^

	Two-stage group (*n* = 20)	One-stage group (*n* = 22)	*P* value
Age, years	52.90 ± 5.87	55.22 ± 6.32	0.165
Sex (male/female)	4/16	6/16	0.580
Dominant/nondominant	6/14	8/14	0.662
Diabetes mellitus	3	2	0.716
Thyroid disease	2	4	0.231
Duration of symptoms, months	11.05 ± 3.74	9.57 ± 2.93	0.152
Thickness of axillary capsule, mm	7.83 ± 2.14	7.36 ± 1.83	0.238
Thickness of coracohumeral ligament, mm	4.25 ± 1.08	3.98 ± 1.42	0.332
Fatty infiltration^b^	0.70 ± 0.57	0.68 ± 0.56	0.912
Tear size, small/medium^c^	3/17	4/18	0.782
Repair technique (single-row/suture bridge)	3/17	4/18	0.782
Concomitant procedures			
Acromioplasty	14	12	0.303
Biceps tenodesis	3	5	0.524
Biceps tenotomy	5	6	0.867
Mean follow-up period, months	25.35 ± 2.13	27.36 ± 4.98	0.102

^a^Values presented as mean ± standard deviation

^b^Graded according to the criteria established by Goutallier and modified by Fuchs

^c^Graded according to the criteria established by DeOrio and Cofield

### Assessment

Demographic data that could affect the outcomes of arthroscopic rotator cuff repair, including patient’s age, sex, hand dominance, diabetes mellitus, thyroid disease, duration of symptoms, fatty infiltration of the rotator cuff muscles, tear size, repair technique, and concomitant procedures (such as biceps tenotomy or tenodesis, acromioplasty, distal clavicle resection), were collected from our database.

### Evaluation of range of motion

For all patients, passive ROM, including forward flexion, external rotation with the arm at the side, and internal rotator, was evaluated preoperatively; 3, 6, 12, and 24 months after surgery; and at final follow-up. Forward flexion and external rotation were evaluated with a goniometer with patients in the supine position. Internal rotation was the highest vertebral level achievable in the midline posteriorly as the patient reached up behind with a “hitch-hiking” thumb. For statistical analysis, internal rotation up to the level of the sacrum was designated as 0 point, and 1 point was added for each level above this. All assessment data were collected by a clinical researcher who was blinded to this study.

### Magnetic resonance imaging (MRI) assessment

All patients underwent MRI (3.0-T MR System, Signa Excite, GE Medical Systems, Waukesha, Wisconsin, USA) examinations preoperatively to evaluate the findings suggestive of adhesive capsulitis, such as thickening of axillary capsule and coracohumeral ligament. Fatty infiltration of the rotator cuff muscle was also evaluated on MRI scan and classified according to the criteria established by Loew et al. [15] and modified by Oh et al. [16]. Scans were evaluated at the level where the scapular spine and body form a Y-shape in the oblique sagittal view. The tear size of rotator cuff was measured intraoperatively under direct arthroscopic visualization with a calibrated probe and classified according to the criteria established by Schmidt and Morrey [17].

### Functional and satisfaction assessments

At final follow-up, functional assessment was performed using visual analogue scale (VAS), American Shoulder and Elbow Surgeons (ASES) score, and Constant-Murley score. VAS was scored on a scale of 0 to 10, with 10 indicating the highest level of pain. The ASES score consisted of a score summation using a 100-point system (50 points for daily function and 50 points for pain). Patients were additionally asked about their satisfaction regarding their clinical outcomes (i.e., very satisfied, satisfied, neutral, or not satisfied). The proportion of very satisfied and satisfied patients was defined as the satisfaction rate.

### Surgical procedure

#### One-stage group

**Fig. 1:**
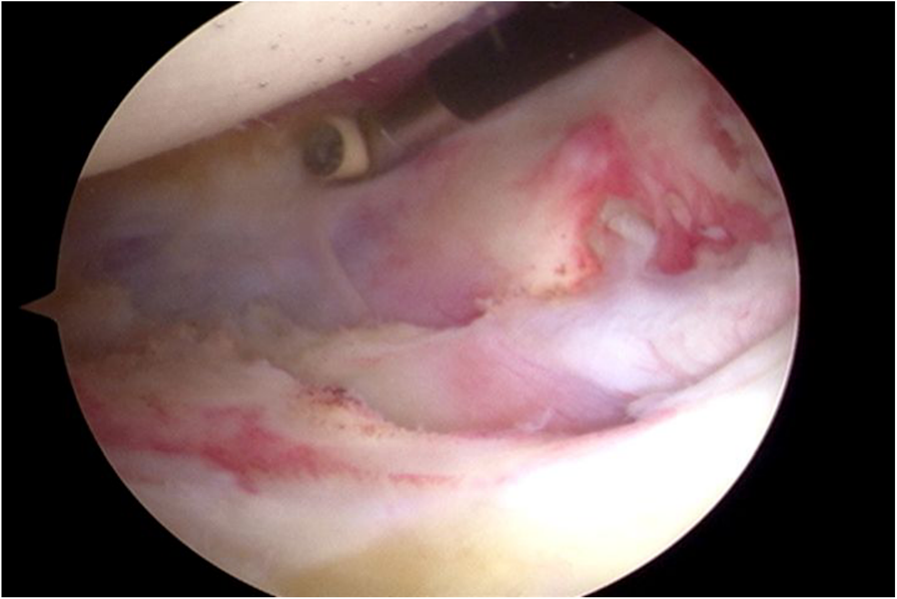
Capsular release was performed using a radiofrequency device

**Fig. 2:**
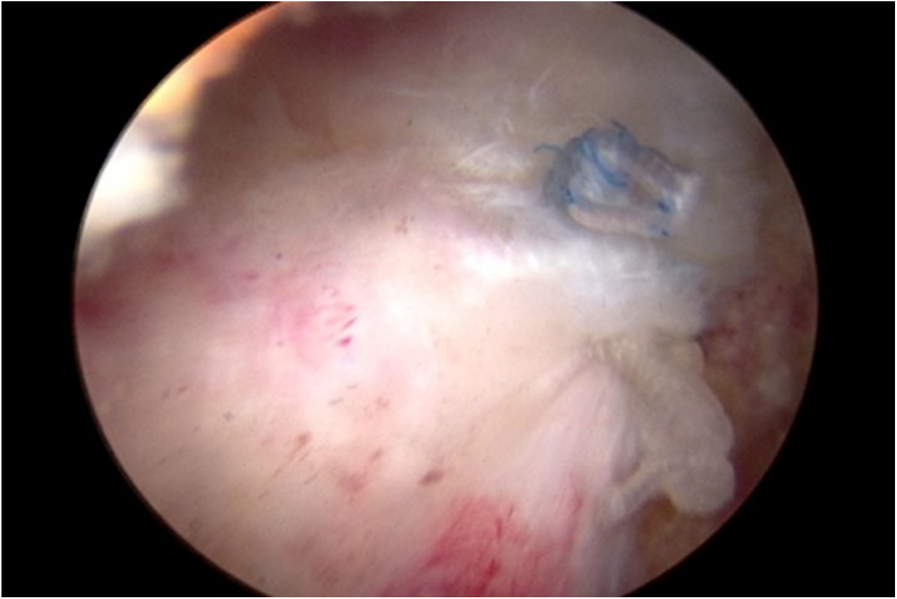
Single-row technique

**Fig. 3:**
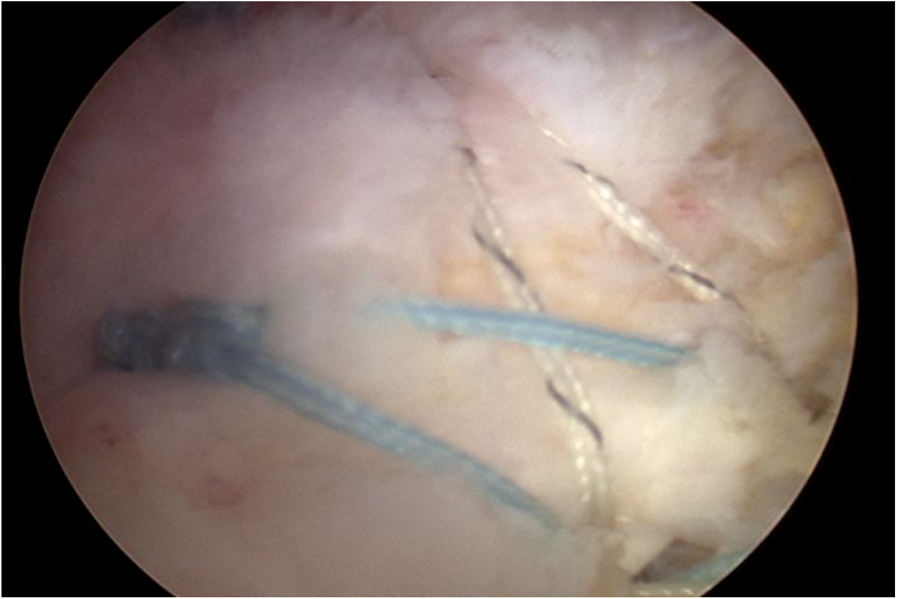
Suture bridge technique

In this group, all the patients underwent a one-stage procedure, including arthroscopic capsule release and concomitant rotator cuff repair within 2 weeks of the diagnosis. After induction of general anesthesia, each patient was positioned in a lateral decubitus position with the involved arm suspended by an arm-holding device using 10 to 15 lb of suspension. A routine arthroscopic glenohumeral examination was performed through the standard posterior and anterior portals. After confirmation of synovial hypertrophy and capsular thickening, sequential release of the rotator interval and anterior, inferior, and posterior capsules using a radiofrequency device (Arthrocare, Sunnyvale, California, USA) was performed (Fig. 1). Capsular release was performed immediately off the glenoid rim to avoid damaging the axillary nerve. The arthroscope was then placed in the subacromial space, and a lateral portal was established as the working portal. After removal of residual bursa and debridement of degenerated tendon edges, the rotator cuff tear was accessed using a calibrated probe. For small-sized rotator cuff tears, repair was conducted via a single-row technique (Fig. 2). For medium-sized rotator cuff tears, a suture bridge technique was applied (Fig. 3).

#### Two-stage group

In this group, conservative treatment for the recovery of ROM was initially performed before arthroscopic rotator cuff repair. Conservative treatment included nonsteroidal anti-inflammatory drugs (NSAIDs), corticosteroid injections, and rehabilitative therapy. Rehabilitative therapy consisted of pendulum circumduction, passive shoulder stretching in forward flexion, external rotation, horizontal adduction, and internal rotation. Patients were instructed to stretch the shoulder to the point of tolerable discomfort and hold the position for 3 s. Rehabilitative therapy was performed thrice every day, and each session lasted at least 15 min. NSAIDs were prescribed when necessary. Rehabilitative therapy was continued for 3 months, and subsequent surgery for rotator cuff repair was performed.

#### Postoperative rehabilitation

The postoperative rehabilitation protocol was identical in both groups. Immobilization was maintained with an abduction brace at 30° for 6 weeks except for showering and rehabilitative therapy. From the first day after surgery, all patients engaged in pendulum, passive forward flexion, and external rotation exercises. Active exercises were not allowed until 6 weeks postoperatively. Muscle strengthening exercises were typically initiated at 3 months postoperatively. A return to recreational activity with heavy demands on the shoulder or to manual labor was delayed for 6 months.

### Statistical analyses

All statistical analyses were performed using SPSS software (IBM-SPSS statistics 19.0; New York, USA). The data were presented as the means and standard deviations for description. Paired *t* test was used to compare the preoperative and postoperative results, including ROM and functional scores. Unpaired *t* test was used to compare the continuous variables between the two groups. Chi-square analyses were used to determine the differences in patient’s sex, side, and satisfaction rate. The significance level was set to 0.05.

## Results

### Preoperative MRI findings

Preoperative MRI revealed thickening of axillary capsule and coracohumeral ligament in all patients (7.58 ± 1.97 mm and 4.11 ± 1.26 mm, respectively). No significant difference was observed between the two groups regarding the thickening of axillary capsule and coracohumeral ligament (7.83 ± 2.14 mm vs. 7.36 ± 1.83 mm, *P* = 0.238; 4.25 ± 1.08 mm vs. 3.98 ± 1.42 mm, *P* = 0.332; respectively, Table 1). The differences in fatty infiltration and rotator cuff tear size were also not significant between the two groups (0.70 ± 0.57 vs. 0.68 ± 0.56, *P* = 0.238; 3/17 vs. 4/18, *P* = 0.782; respectively, Table 1).

### Range of motion

**Fig. 4:**
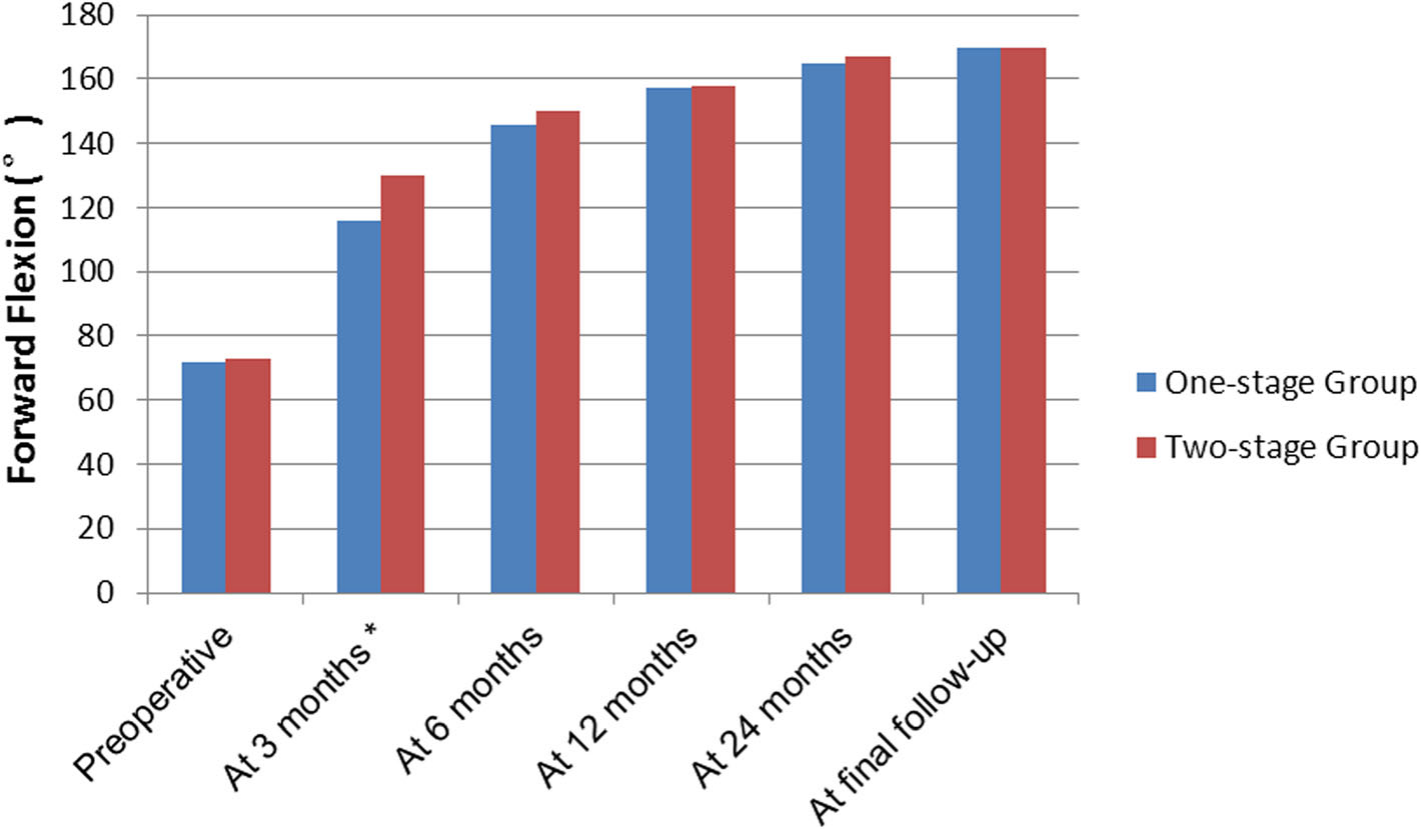
The mean changes in forward elevation. At 3 months postoperatively, the two-stage group exhibited significantly improved forward flexion compared with the one-stage group (*P* = 0.001). No significant differences between groups were noted at any other time point (*P* > 0.05)

**Fig. 5:**
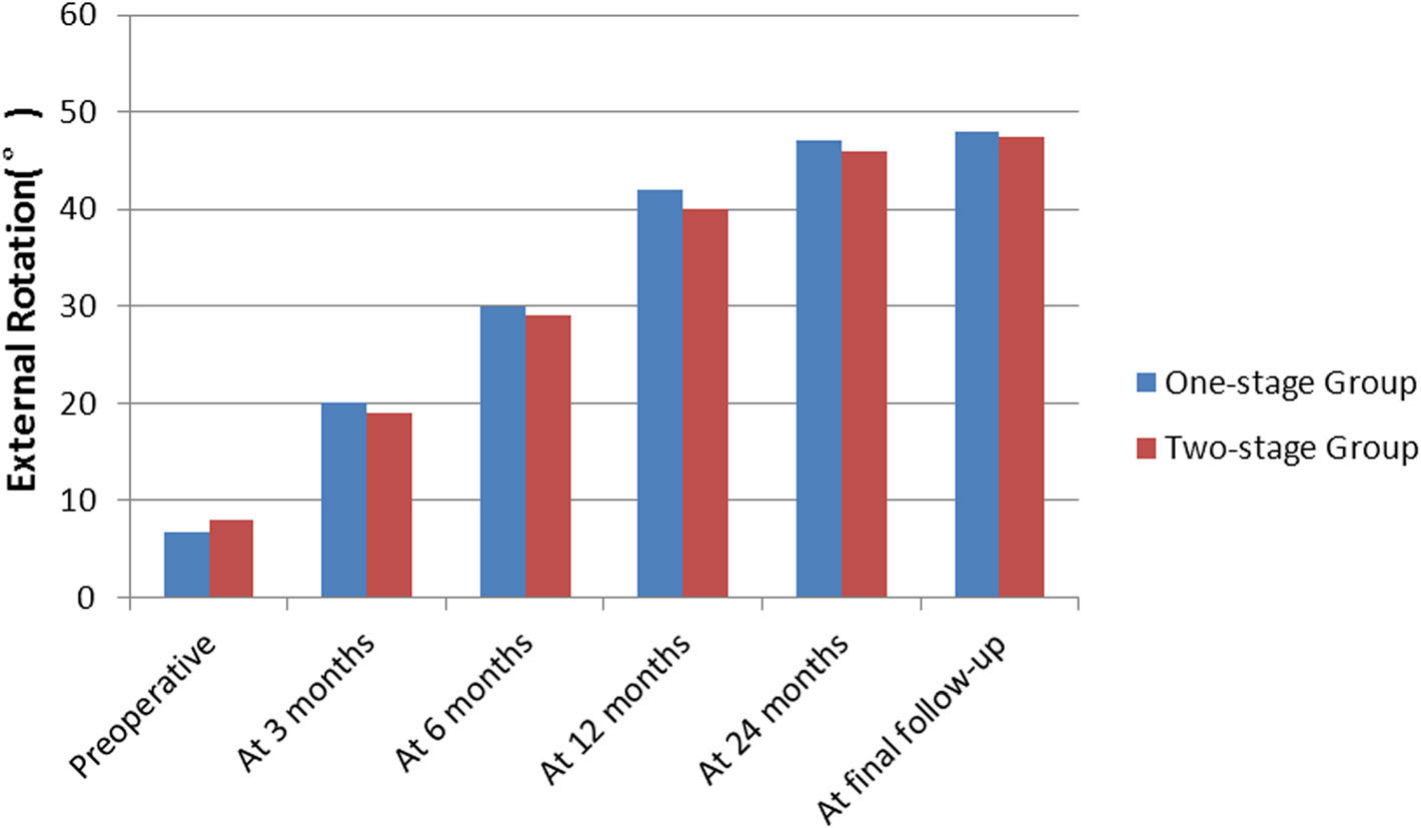
The mean changes in external rotator. No significant differences between groups were noted at any time point (*P* > 0.05)

**Fig. 6:**
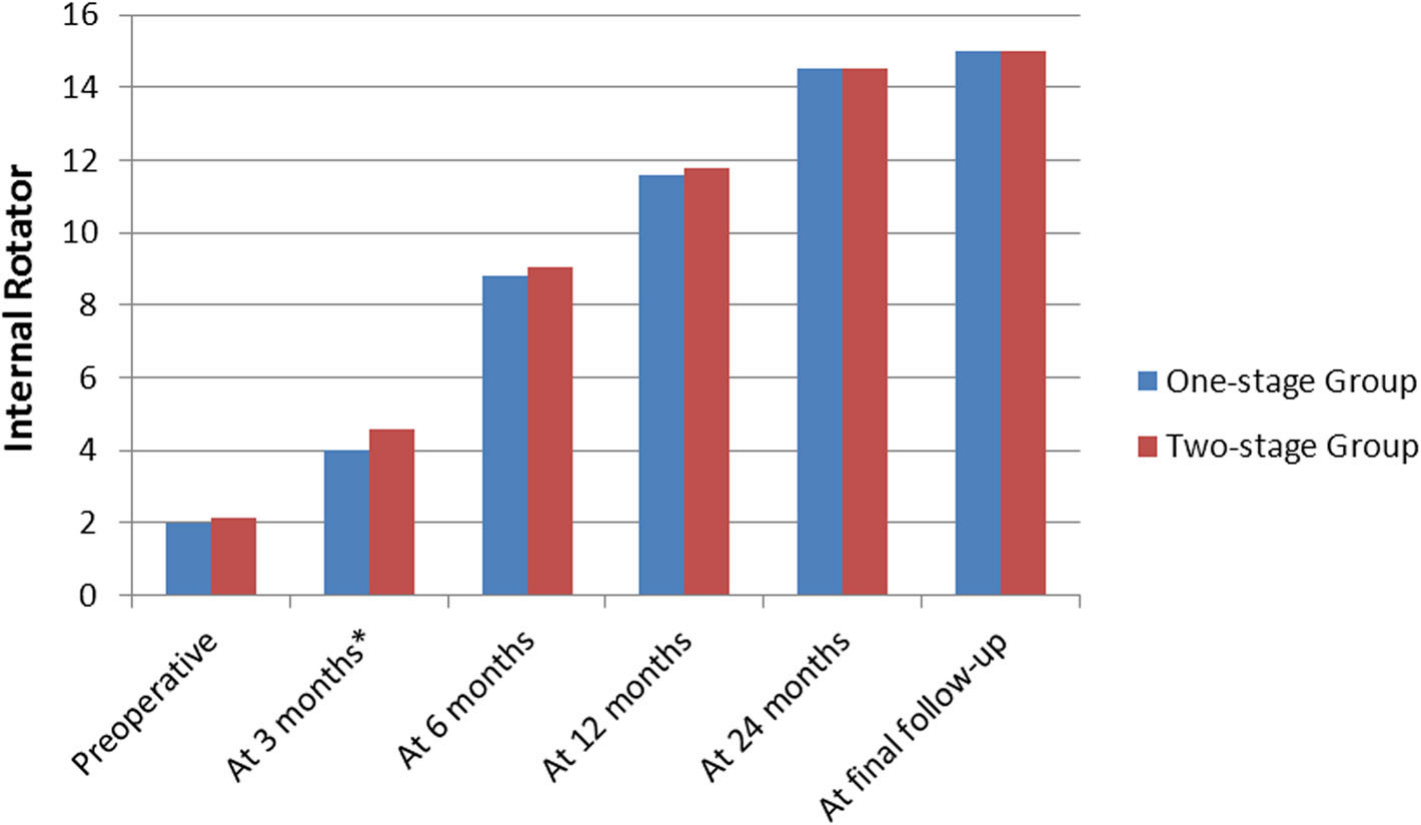
The mean changes in internal rotator. At 3 months postoperatively, the two-stage group exhibited significantly improved internal rotator compared with the one-stage group (*P* = 0.038). No significant differences between groups were noted at any other time point (*P* > 0.05)

Before treatment, no significant difference in ROM was observed between the two groups (*P* > 0.05, Figs. 4, 5, and 6). In two-stage group, six patients withdrew from rehabilitative therapy due to severe pain and underwent surgery in advance. However, the remaining 14 patients exhibited significantly improved forward flexion and internal rotation after rehabilitative therapy (72.50° ± 10.35° to 104.50° ± 12.90°, *P* < 0.05; 2.07 ± 0.98 to 3.15 ± 0.86, *P* < 0.05). Two patients did not undergo subsequent surgery due to significant improvement in pain relief and ROM.

At 3 months postoperatively, the two-stage group exhibited significantly improved forward flexion and internal rotation compared with the one-stage group (*P* = 0.001 and *P* = 0.038, respectively, Figs. 4 and 6). However, the difference in external rotation between the two groups was not significant (*P* > 0.05, Fig. 5). No significant difference in ROM was noted between the two groups at 6, 12, and 24 months and final follow-up (*P* > 0.05). In addition, both groups achieved significantly improved ROM at the final follow-up compared with ROM before treatment (Figs. 4, 5, and 6).

### Functional outcomes

Both groups exhibited significant improvements in the VAS score, ASES score, and Constant score at final follow-up (*P* = 0.001). No significant difference was observed between the two groups regarding the VAS score, ASES score, and Constant score at any period after surgery (*P* > 0.05, Table 2). The satisfaction rate was 90.5% in the one-stage group and 68.2% in the two-stage group. No significant difference in satisfaction rate was noted between the two groups (*P* = 0.085, Table 2).

**Table 2:** Comparison of clinical outcomes between the two groups

	Two-stage group (*n* = 20)	One-stage group (*n* = 22)	*P* value
VAS			
Preoperative	5.60 ± 0.75	5.72 ± 0.93	0.632
At final follow-up	1.65 ± 0.74	1.40 ± 0.79	0.319
*P* value	0.001	0.001	
ASES			
Preoperative	41.95 ± 10.58	40.10 ± 9.61	0.557
At final follow-up	81.27 ± 6.94	83.75 ± 8.11	0.529
*P* value	0.001	0.001	
Constant-Murley			
Preoperative	36.85 ± 8.08	39.40 ± 6.59	0.266
At final follow-up	79.50 ± 7.77	78.59 ± 7.95	0.711
*P* value	0.001	0.001	
Satisfaction rate	90.0%	68.2%	0.085

*Abbreviations*: *VAS* visual analogue scale, *ASES* American Shoulder and Elbow Surgeons score

## Discussion

The principal findings of the present study were that overall satisfactory clinical outcomes could be achieved in both patients after a one-stage procedure or two-stage procedure for rotator cuff tear and concomitant shoulder stiffness. At 3 months, the two-stage group exhibited significantly improved forward flexion and internal rotator compared with the one-stage group. However, no significant differences in ROM were noted between the two groups at any other time point (*P* > 0.05). In addition, no significant differences in functional scores and satisfaction rate were noted between the two groups at final follow-up (*P* > 0.05).

### Factors leading to shoulder stiffness in patients with rotator cuff tear

Several studies have reported that patients with rotator cuff tears could also have shoulder stiffness [3, 4]. Chuang et al. [3] reported on a cohort of 72 patients who underwent rotator cuff repair and found that 40% of the patients exhibited concomitant shoulder stiffness. According to the published literature [3–5, 11, 18], factors leading to shoulder stiffness in patients with rotator cuff tear include the following: (1) the pain from rotator cuff tears results in joint disuse, contracture of the joint capsule, and secondary muscular weakness, which would ultimately facilitate joint stiffness; (2) secondary adhesive capsulitis, which is precipitated by inflammation from the rotator cuff tear, could also contribute to joint stiffness.

Evidence also suggests that many dismetabolic diseases are associated with rotator cuff tear and adhesive capsulitis, such as diabetes mellitus, thyroid disease, Dupuytren’s contracture, and cardiorespiratory and autoimmune diseases [19–22]. Oliva et al. demonstrated a significant role for thyroid hormones in modifying and increasing the rate of non-traumatic rotator cuff tear [21]. Zreik et al. reported that diabetic patients were fivefold more likely to develop adhesive capsulitis compared with non-diabetic controls [20]. Schiefer et al. reported a significant increased prevalence of hypothyroidism in the adhesive capsulitis group compared with the control group (27.2% vs. 10.7%; *P* = 0.001) [22].

### Management of patients with rotator cuff tear and concomitant stiffness

The optimal management of patients with rotator cuff tears and concomitant shoulder stiffness still remains controversial [5–14]. The main concern about the one-stage procedure is the high risk of developing postoperative stiffness [8–10]. Hsu et al. [10] reported on a cohort of 489 patients who underwent rotator cuff repair. In total, 24 patients (4.9%) developed postoperative stiffness, and patients with preoperative shoulder stiffness were associated with a significantly increased incidence of 15.6% for postoperative stiffness. In the current study, we found that patients who underwent the one-stage procedure exhibited significantly reduced forward flexion and internal rotator at 3 months postoperatively.

Nevertheless, the two-stage procedure also has its own inherent disadvantages, including the following: (1) the two-stage procedure would prolong the patient’s suffering given the delay in surgery. In the current study, six patients refused to tolerate the remaining rehabilitation period due to severe pain during stretching exercises. In another study by Huberty et al. [11], the author also reported on a series of 33 patients who underwent conservative treatment before rotator cuff repair, and six patients (18.2%) withdrew due to severe pain during rehabilitation; (2) nonsurgical treatment for shoulder stiffness may be insufficient, especially in the presence of rotator cuff lesions [23–25]; (3) in addition, inappropriate exercise could lead to fatigue accumulation in the damaged tendon, which could potentially exacerbate the rotator cuff injury [11].

### The clinical outcomes of surgical treatment for rotator cuff tear and concomitant shoulder stiffness

Recently, several studies have reported overall satisfactory clinical outcomes of surgical treatment for rotator cuff tears and concomitant stiffness [5, 6, 9–12]. Goutallier et al. [8] reported on a cohort of 211 patients who underwent rotator cuff repair. Forty-three patients exhibited severe concomitant shoulder stiffness and underwent one-stage arthroscopic capsular release and rotator cuff repair. The clinical outcomes of the stiffness group were statistically the same as those in the non-stiffness group. Kim et al. [12] reported a retrospective comparative study of 125 patients who underwent rotator cuff repair. Thirty patients exhibited concomitant moderate shoulder stiffness at the time of the repair. They found that differences in ROM and functional scores did not reach statistical significance 6 months after the operation if arthroscopic capsular release with manipulation is added to the cuff repair procedure. DeOrio and Cofield [5] reported on a cohort of 45 patients and also determined good clinical outcomes after rotator cuff repair with concomitant manipulation for treatment of rotator cuff tears with stiffness.

However, the weakness of these studies is the lack of data about the clinical outcomes after the two-stage procedure for treatment of rotator cuff tears and concomitant stiffness, which makes it impossible to determine whether the one-stage procedure or two-stage procedure is associated with better clinical outcomes.

Recently, Huberty et al. [11] reported on a cohort of 63 patients with rotator cuff tears and stiffness to compare the clinical outcomes of immediate rotator cuff repair with capsular release with patients undergoing rotator cuff repair after the stiffness was treated with rehabilitative therapy. The author reported improved results in both groups after 6 months postoperatively, and the effect was maintained until 12 months postoperatively. In the present study, we further confirmed that similar satisfactory clinical outcomes could be maintained until 24 months postoperatively.

### Limitations

There are some limitations to our study. First, this was a retrospective study that included all of the inherent limitations of this study design. Second, our study involved a relatively small number of patients. Third, the length of the follow-up was relatively short, and longer-term evaluations are required to compare the clinical outcomes of one-stage and two-stage procedures for rotator cuff tear and concomitant stiffness.

## Conclusion

Regarding the treatment of rotator cuff tear with concomitant stiffness, although the recovery of ROM took longer in patients who underwent the one-stage procedure, satisfactory results at final follow-up can be achieved using either the one-stage procedure or two-stage procedure. To avoid unnecessary rehabilitation, the one-stage procedure may be a helpful option for patients with rotator cuff tear and concomitant stiffness.

## Abbreviations

ASES: American Shoulder and Elbow Surgeons score
NSAIDs: Nonsteroidal anti-inflammatory drugs
ROM: Range of motion
VAS: Visual analogue scale
